# Supplementary material for: Quantum enhanced radio detection and ranging with solid spins
Source: Nat Commun. 2023 Mar 9;14:1288. doi: 10.1038/s41467-023-36929-8 (PMC9998632; doi:10.1038/s41467-023-36929-8)
Supplement: Supplementary file 1 — Supplementary Information [file 41467_2023_36929_MOESM1_ESM.pdf]

Supplementary Information for:

"Quantum enhanced radio detection and ranging with solid spins"

Xiang-Dong Chen[1,2,3], En-Hui Wang[1,2], Long-Kun Shan[1,2], Shao-Chun Zhang[1,2,3], Ce Feng[1,2,3], Yu Zheng[1,2], Yang Dong[1,2,3], Guang-Can Guo[1,2,3], Fang-Wen Sun[1,2,3]

[1] *CAS Key Laboratory of Quantum Information, School of physics, University of Science and Technology of China, Hefei, 230026, People's Republic of China.*

[2] *CAS Center For Excellence in Quantum Information and Quantum Physics, University of Science and Technology of China, Hefei, 230026, People's Republic of China.*

[3] *Hefei National Laboratory, University of Science and Technology of China, Hefei, 230088, People's Republic of China*

Supplementary Note 1. The amplitude sensitivity of RF field

By detecting the spin-dependent fluorescence with a green laser pulse, the Rabi oscillation of NV center is observed as:

$$I = \frac{I_0 + I_1}{2} + \frac{I_0 - I_1}{2} e^{-t_{\mathrm{RF}}/\tau} \cos(\Omega t_{\mathrm{RF}}), \tag{1}$$

where $I_0(I_1)$ depicts the fluorescence intensity with $m_{\mathrm{s}} = 0$ ($m_{\mathrm{s}} = \pm 1$). The response of NV center fluorescence to the RF field is

$$\frac{dI}{dB_{\mathrm{RF}}} = \frac{I_0 - I_1}{2} e^{-t_{\mathrm{RF}}/\tau} 2\pi\gamma k t_{\mathrm{RF}} \sin(\Omega t_{\mathrm{RF}}). \tag{2}$$

The local maximum will be obtained at $\mid \sin(\Omega t_{\mathrm{RF}}) \mid = 1$. The oscillation amplitude is written as $I_0 - I_1 \propto C\varepsilon t_{\mathrm{det}}$, where C represents the fluorescence contrast ratio between different spin states, ε is the fluorescence counting rate and t_{det} is the spin detection time. The Rabi oscillation is enhanced as $\Omega = k \times 2\pi\gamma B_{\mathrm{RF}}$.Then, the maximum optical responsibility is $dI/dB_{\mathrm{RF}} \propto C\varepsilon t_{\mathrm{det}} \gamma k t_{\mathrm{RF}} e^{-t_{\mathrm{RF}}/\tau}$.

The magnetic sensitivity of the free space RF field is given by the minimum detectable RF field variation σ_{B} in a total measurement time T, as $\eta_{\mathrm{B}} = \sigma_{\mathrm{B}}\sqrt{T}$. The measurement time includes the RF-spin interaction time t_{RF} and the spin detection time t_{det}. By recording the fluorescence of NV center, the minimum detectable RF field is deduced from the optical signal's deviation σ_{I} and the optical response to RF field, as $\sigma_{\mathrm{B}} = \frac{\sigma_{\mathrm{I}}}{dI/dB_{\mathrm{RF}}}$. Determined by the shot noise, the deviation of the optical signal will scale as $\sigma_{\mathrm{I}} \propto \sqrt{\varepsilon t_{\mathrm{det}}}$. Therefore, the magnetic sensitivity of the free space RF field will be

$$\eta_B \propto \frac{1}{C\sqrt{\varepsilon t_{\mathrm{det}}}} \frac{1}{\gamma k} \frac{1}{e^{-t_{\mathrm{RF}}/\tau}} \frac{1}{t_{\mathrm{RF}}} \sqrt{t_{\mathrm{RF}} + t_{\mathrm{det}}}. \tag{3}$$

It indicates that the sensitivity is determined by the fluorescence intensity, the local spin-RF interaction strength, the spin decoherence time and the spin-RF interaction time. In the experiment, the spin detection time t_{det} is approximate 1 μs, longer than the spin-RF interaction time and the Rabi oscillation decay time τ. The highest sensitivity would be obtained with $t_{\mathrm{RF}} \approx \tau$.

Supplementary Note 2. The enhancement of local RF-spin interaction

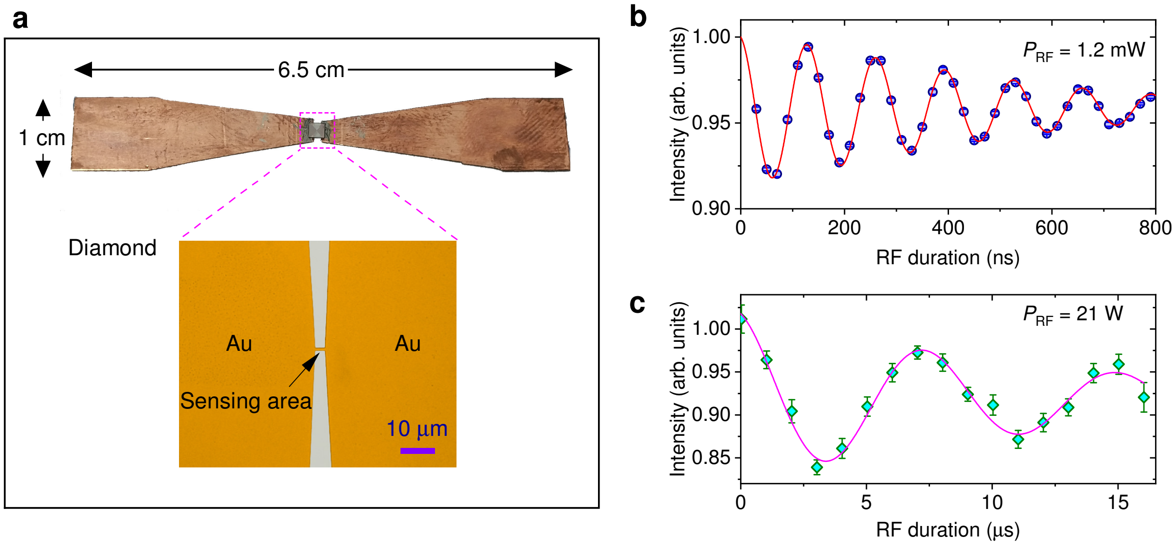

Supplementary Figure 1. **Enhancement of RF-Spin interaction at nanoscale. a** Photographs of the whole nanowire-bowtie structure. The two arms of bowtie antenna is connected by a wire with a width of approximately 1 μm. The spin-RF interaction is measured near the nanowire. **b**, **c** The Rabi oscillation of NV center electron spin with and without the nanowire-bowtie structure, respectively. $P_{\rm RF}$ is the power of free space RF that is emitted by the horn antenna. Error bars in (**b**, **c**)represent the standard errors of measurements.

A nanowire-bowtie structure is used to enhance the RF-spin interaction at nanoscale. The configuration of the RF antenna is shown in Supplementary Fig. 1a. A small nanowire-bowtie structure of Au film is first produced on the diamond surface via photolithography. It is subsequently connected to a larger bowtie antenna of copper tap. The fluorescence of NV center, which is near the nanowire, is detected with a confocal microscope.

To quantify the enhancement of the local RF-spin interaction, we measured the Rabi oscillation of NV center with and without nanowire-bowtie structure. As shown in Supplementary Fig. 1b, excited by a 1.2 mW free space RF field, a Rabi oscillation frequency of 7.5 MHz is observed with the antenna. In contrast, the Rabi oscillation frequency is only 0.13 MHz with the direct pumping of a 21 W free space RF field (Supplementary Fig. 1c). Since the Rabi oscillation frequency is proportional to the square root of RF power, we estimate that the local RF-spin interaction is enhanced approximately $k = 7.6\times\ 10^3$ times.

Supplementary Note 3. The saturation of photon detector

In this work, we used a single-photon-counting-module (SPCM-AQRH-15-FC, Excelitas) to detect the fluorescence signal of NV center. The saturation of SPCM with a high counting rate will decrease the detected spin state contrast C, as indicated in Supplementary Fig. 2. To reduce the saturation of SPCM, we placed a neutral density (ND) filter with OD = 1 in the fluorescence collecting path. In future applications, the saturation problem can be solved by using a photon detector with a higher dynamic range.

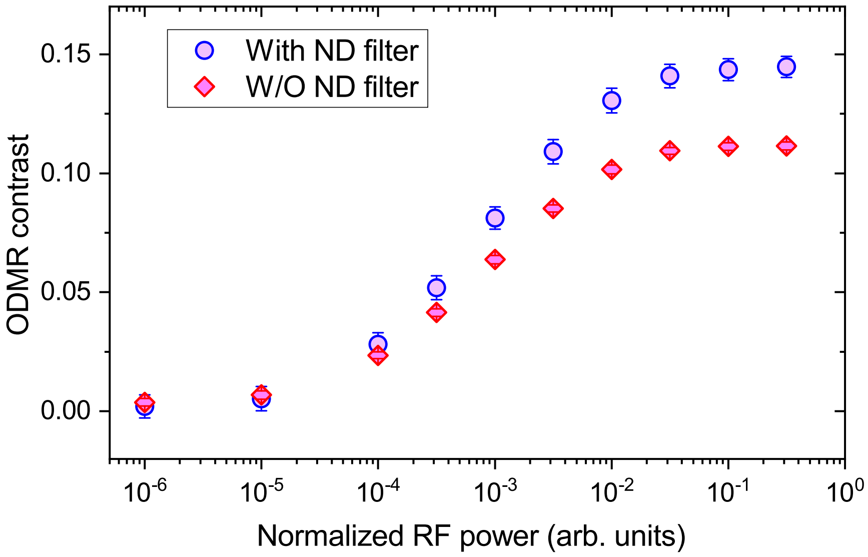

Supplementary Figure 2. **The SPCM saturation decreases the ODMR contrast.** The CW-ODMR signal of NV center is recorded with a 0.17 mW green laser pumping. The fluorescence counting rates with and without the ND filter are 1.2 and 7.6 Mcounts/s, respectively. The saturated counting rate of SPCM is 35 Mcounts/s. The results show that the ODMR contrast is decreased with a high counting rate. Error bars represent the standard errors of measurements.

Supplementary Note 4. Rabi oscillation with different RF powers

For Rabi oscillation of high density NV center ensemble, both the decay time and amplitude of oscillation will change with RF power. In Supplementary Fig. 3, we measure the Rabi oscillation of NV center in our diamond sample with Rabi frequency from 0.4

to 8.4 MHz. It shows that the decay time decreases with Rabi frequency. Meanwhile, the amplitude of Rabi oscillation increases with Rabi frequency, due to the inhomogeneous broadening. Overall, the response of NV center ensemble to RF magnetic field will increase with the Rabi frequency. It indicates that a high RF sensitivity should be obtained with a relative high RF power. In future applications, a strong bias RF field can be applied to solve this problem.

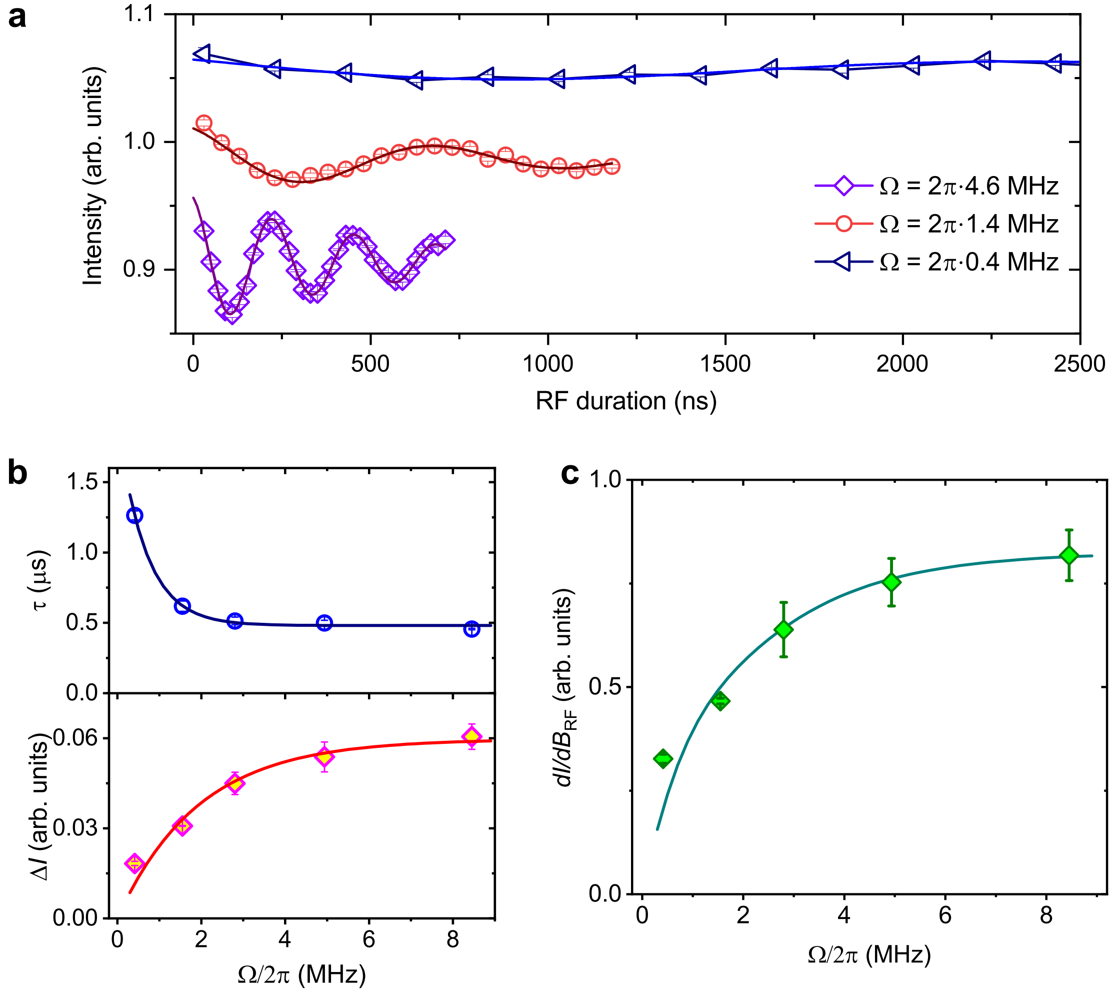

Supplementary Figure 3. **The contrast of Rabi oscillation changes with the RF excitation.** **a** The Rabi oscillation of NV center ensemble with different excitations strengths. **b** The decay time (τ) and amplitude (ΔI) of oscillation changes with Rabi frequency. They are obtained by fitting the Rabi oscillation results with an exponentially damped cosine function. **c** The estimated optical response of NV center with different Rabi frequencies. The RF pulse duration time was set to 200 ns in (**c**). Error bars represent the standard errors of measurements.

Supplementary Note 5. FWHM of ranging signal

According to Eq. (3) in the main text, the FWHM of ranging signal will be determined by the distance of two neighboring positions, where $\Omega t_{\text{RF}} = 0$ and $\pi/2$, respectively. Substituting in Eq.(2) of main text, we deduce that $\Omega t_{\text{RF}} = 0$ will be obtained at a position $L_1 = \frac{1}{2}(n + \frac{1}{2})\lambda$, where n is the integer ambiguity. And $\Omega t_{\text{RF}} = \pi/2$ can be obtained at the position L_2, where $\cos(2\pi\frac{L_2}{\lambda}) = \pm\frac{1}{2N}$. Here, we have used the definition of $N = 4k\gamma B_1 t_{\text{RF}}$. Then, we get

$$\cos(n\pi + \frac{\pi}{2} + 2\pi\frac{\Delta L}{\lambda}) = \pm\frac{1}{2N}, \quad (4)$$

where $\Delta L = L_2 - L_1$. It is simplified as $sin(2\pi\frac{\Delta L}{\lambda}) = \pm\frac{1}{2N}$. For $\Delta L \ll \lambda$ in our experiments, the equation is approximated as

$$2\pi\frac{\Delta L}{\lambda} \approx \pm\frac{1}{2N}. \quad (5)$$

It shows that, the FWHM of ranging signal will be changed as $\text{FWHM} = 2|\Delta L| = \frac{\lambda}{2N\pi}$.

Supplementary Note 6. Time of flight

The time of flight (ToF) can be estimated using the pulse sequence in Supplementary Fig. 4a. The temporal overlap between reference and backscattered RF pulses changes with the ToF, and determines the intensity of interference. As an example, we set the phase difference between the reference and backscattering to π. Then, the amplitude of localized RF, which interacts with NV center, will be reduced with the temporal overlap. It subsequently decreases the spin transition rate. We see that the fluorescence intensity of NV center changes with the ToF of backscattered RF in Supplementary Fig. 4b. Compared with the noise ratio (as depicted in the main text), we deduce that the ToF can be measured with an accuracy of approximately 0.5 ns (with a measurement time of 1 s). It corresponds to a ToF ranging accuracy of 7.5 cm. The sampling rate and accuracy could be further enhanced by increasing the fluorescence counting rate in future applications.

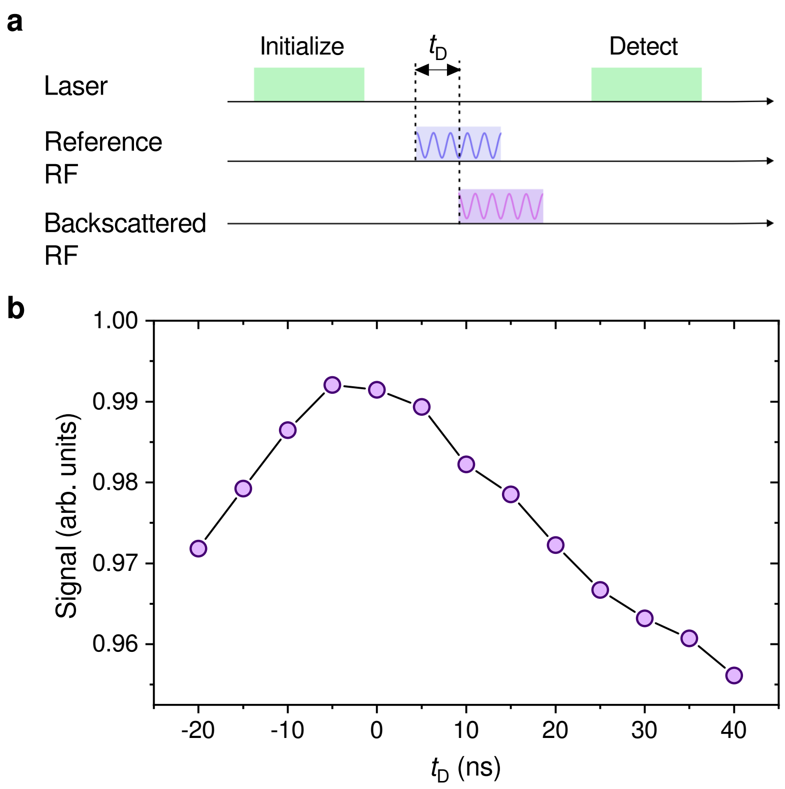

Supplementary Figure 4. **ToF measurement with NV center.** **a** The laser and RF pulse sequences for ToF ranging. t_{D} represents the time delay between scattered and reference RF pulses. **b** The spin-dependent fluorescence of NV center changes with the delay between backscattered and reference RF pulses. The RF peak power for ranging is approximately 0.35 mW. The width of RF pulse is 30 ns. The maximum optical response to the delay time of back scattering is measured to be approximately 0.13%/ns.
